# Repression of Illegitimacy

**Published:** 1859-09

**Authors:** 


### *Repression of Illegitimacy.*

Our civilization is fairly open to the reproach of fastidious prudery and excess of delicacy. The kid glove school of social reformers shrink from handling the uncleau sores that affect the body corporate of humanity and have always preferred that they should be swathed in the manifold bandages of conventional decency, plastered with the old fashioned unguents of mechanical charity, and left to the care of the providence that guards unseen misery and misfortune. It is not suprising, therefore, that the active advocates of social progress, who come now to rid us of reproach of a too delicate abstinence from the investigation of such evils, should find that materials are wanting even for the first stage of the enquiry. The great element in an investigation which aims at reform is an accurate estimate of the evil to be reformed. Amongst the defects of our society which most constantly obtrusively present themselves, is illegitimacy; an evil which haunts all our parishes, in town or country; which rears its head in every hamlet, and stains the purity of every village. It lurks in the smiling corn fields, and in the dark alleys of the crowded town. Amid the Presbyterian population of Scotland, and the Catholic people of Austria, it attains a higher fruition of sin and distress than in any other countries of Europe. In the kingdom of great Britain some 45,000 illegitimate children are annually born. There are many circumstances of singular and contradictory import connected with the advent of these unfortunates, of whom so many are destined to an early death, from neglect, cold, desertion, starvation and violence. The proportion of illegitimate births was found by the registrar general of Scotland to be greatest, not amongst the seats of rapidly advancing population, or in those counties which contain our largest cities with their overcroweded inhabitants, but in those which are purely agricultural. The vastness of the evil, and its surprising excess in some localities when compared with others, might well introduce a careful investigation of all that relates to its growth, or may be supposed to favor its extention. Yet Mr. Acton stated last week, in a paper which he read before the Statistical society, "On illegitimacy in the London parishes of St. Marylebone, St. Pancras, St. George, Southwark," that illegitimacy has no literature; and in looking through the lately published catalogue of the Statistical and other societies, he failed to find mention of the word. Mr. Acton made a very able and useful contribution to the study of this important question by the analysis of the materials existing in these three extensive parishes. Analyzing the published figures of the registrar general, he showed that in 1857, out of 388 illegitimate children who died, 327 fell before attaining the age of one year, of whom 110 perished between the ages of one and three months. Few children died within the first week of birth; hence it may be concluded that they are born healthy, and that the excessive mortality is due to neglect, probably consequent on the destitution of the mother.

Surely this sequence of facts appeals loudly to the charity of the worldly prosperous, that they dismiss the reluctance to assist woman who have given birth to illegitimate children, not only out of mercy to their fallen condition, but in pity for the young lives that hang upon them. One hundred and ninetyfour mothers were domestic servants; as to the occupation of the fathers, it appears that the largest number are of the class of laborers, where the source of evil to be removed is the promiscuous herding of both sexes, so common among the poorer classes; and next rank domestics, indicating a cause of immorality already sufficiently known.

Mr. Acton indicates striking defacts in the bastardy laws, and suggests that parishes should have the same power of recovering the sums expended on illegitimate children as they have now from the fathers of those born in wedlock. To cut off the supply of harlots, he suggests that the demand should be diminished, by making the penalties in purse and person heavier than they now are, as against the father of the child. Hitherto, suggestions have been mainly confined to the regulation of the sources of the supply. Mr. Acton aimsat checking the demand. It is a maximum of approved force in economic science, and we see no reason why it should not be brought into play. Let the fathers be legally liable to the parish for the expense of the accouchement of the woman and the rearing of the child, and let the parish be armed with power to recover the amounts so expended. Of course no profits should accrue to the mother as the wages of her sin; for the term " seducer," so constantly applied to her paramour, is something more than a mere conventionality—it is very often a falsehood. Such provisions would undoubtedly increase the number of marriages amongst those who have mated irregularly, being equal in rank of life; and they would, we believe, greatly repress the evil discussed. Only let us beware of encouraging the action of government boards, such as Mr. Acton suggests; this were an infallible recipe for bringing things to a deadlock.—*Va Med. Journal.*

